# Association Between Vitamin D Supplementation and Fall Prevention

**DOI:** 10.3389/fendo.2022.919839

**Published:** 2022-08-10

**Authors:** Fei-Long Wei, Tian Li, Quan-You Gao, Yuli Huang, Cheng-Pei Zhou, Wen Wang, Ji-Xian Qian

**Affiliations:** ^1^ Department of Orthopedics, Tangdu Hospital, Fourth Military Medical University, Xi’an, China; ^2^ School of Basic Medicine, Fourth Military Medical University, Xi’an, China; ^3^ The George Institute for Global Health, Faculty of Medicine, University of New South Wales, Sydney, NSW, Australia; ^4^ Department of Cardiology, Shunde Hospital, Southern Medical University, Foshan, China; ^5^ Department of Radiology and Functional and Molecular Imaging Key Laboratory of Shanxi Province, Tangdu Hospital, Fourth Military Medical University, Xi’an, China

**Keywords:** vitamin D, fall, prevention, association, risk

## Abstract

**Background:**

Falls occur frequently among older individuals, leading to high morbidity and mortality. This study was to assess the efficacy of vitamin D in preventing older individuals from falling.

**Methods:**

We searched the PubMed, Cochrane Library, and EMBASE databases systematically using the keywords “vitamin D” and “fall” for randomized controlled trials (RCTs) comparing the effects of vitamin D with or without calcium supplements with those of a placebo or no treatment on fall incidence in adults older than 50 years. A meta-analysis was performed to calculate risk ratios (RRs), absolute risk differences (ARDs) and 95% CIs with random-effects models.

**Results:**

A total of 38 RCTs involving 61 350 participants fulfilled the inclusion criteria. Compared with placebo, high-dose vitamin D (≥ 700 IU) can prevent falls [RR, 0.87 (95% CI 0.79 to 0.96); ARD, -0.06 (95% CI, -0.10 to -0.02)]. Low-dose vitamin D (<700 IU) was not significantly associated with falls. Subgroup analysis showed that supplemental calcium, 25(OH) D concentration and frequency influenced the effect of vitamin D in preventing falls. Sensitivity analysis showed that vitamin D prevented falls, which was consistent with the primary analysis. In addition, the active form of vitamin D also prevented falls.

**Conclusion:**

In this meta-analysis of RCTs, doses of 700 IU to 2000 IU of supplemental vitamin D per day were associated with a lower risk of falling among ambulatory and institutionalized older adults. However, this conclusion should be cautiously interpreted, given the small differences in outcomes.

**Systematic Review Registration:**

https://www.crd.york.ac.uk/prospero/, identifier CRD42020179390.

## Introduction

Falls are the leading cause of accidental injuries and fractures in the elderly (1). One out of every three people over 65 years of age has experienced at least one fall (2), and approximately 20% of the falls required medical attention (2). Globally, approximately 684,000 people die from falls each year, more than 80% of which occur in low- and middle-income countries (3). In 2019, the incidence rate of falls among people aged 60 years and older was 3799.4 new falls per 100 000 population in China (4). Therefore, prevention of falls is widely regarded as the most important element in injury and fracture prevention plans for older individuals.

Vitamin D has a direct influence on muscle strength and is regulated by specific vitamin D receptors in muscle tissue (5). Insufficient vitamin D is associated with lower physical performance and greater declines in physical functioning (6, 7). And vitamin D deficiency can lead to secondary hyperparathyroidism, increased bone resorption, decreased bone mineral density (BMD) and the consequent increase of fracture risk. In some studies of older people at risk of vitamin D deficiency, vitamin D supplements can improve strength, function, and balance., which resulted in a reduction in falls (6, 8). However, the meta-analyses of clinical trials have not found the role of vitamin D in reducing falls. The vitamin D supplement intervention has mixed results on all aspects of prevention (2, 9–11).

Older people living in nursing homes are more likely to fracture than people living in the community (12). However, it is not clear whether life dwelling affect the role of vitamin D in preventing falls. Previous studies have not distinguished the impact of vitamin D on different populations (2, 10, 13). Whether taking calcium affects falling is still uncertain. Therefore, we conducted this meta-analysis to evaluate the effectiveness of vitamin D in preventing falls.

## Methods

This meta-analysis is based on the Cochrane Handbook for Systematic Reviews of Interventions (14) and the Preferred Reporting Items for Systematic Reviews and Meta-analyses guidelines (15, 16). The protocol was published in PROSPERO (CRD42020179390).

### Data Sources and Searches

A systematic online search was performed for eligible trials using the electronic databases PubMed, Embase and the Cochrane Library from their inception dates to February 15, 2020, to identify recently published randomized controlled studies (RCTs) assessing the relationship between vitamin D (with or without calcium) and the incidence of falls (search strategies are reported in **eTable 1**). The initial searches were updated on May 10, 2020. Two authors worked independently (F-L W, T L).

### Study Selection

Each study’s abstract and full text was reviewed by two reviewers (F-L W, T L) independently to determine eligibility. Conflicts were resolved through discussion. RCTs were selected based on the following inclusion criteria (1): Studies comparing vitamin D or combination of vitamin D and calcium with no placebo or treatment (2); RCTs including adults aged 50 years old or older; and (3) trials providing fall data. The exclusion criteria were as follows (1): RCTs with no placebo or no treatment group (2); observational or animal studies (3); studies for stroke patients, organ transplant patients, or parkinson patients (4) RCTs that evaluated intramuscular injection of vitamin D. Only those trial designs that were double-blind and fully assigned an evaluation of falls were included in the primary analysis: (a) falling was the main outcome; (b) the study should clarify the definition of a fall and its assessment; and (c) falling must be evaluated throughout the study. Otherwise, trials were included in the sensitivity analysis.

### Data Extraction and Quality Assessment

Our primary outcome was the relative risk of a person who had at least one fall and took vitamin D supplements compared with a person who took a placebo or calcium supplements alone. The effects of supplemental vitamin D and active forms of vitamin D were analyzed separately.

Data were independently extracted by two researchers (F-L W, T L). The informations obtained from each study were as follows: year of publication; first author; country of origin; characteristics of participant; calcium and vitamin D doses, alone or combination; serum 25-hydroxyvitamin D concentration; and duration. We only extracted the relevant data.

The methodological quality of the included RCTs was independently evaluated by two authors (F-L W, T L). Disagreements were resolved through consensus. According to Cochrane’s bias risk criteria, Each quality item was classified as low, high, or undefined risk (14, 17). Trials with dissimilar baseline characteristics between different intervention groups were considered to have other bias.

### Data Synthesis and Analysis

The researchers evaluated the effects of vitamin D supplementation and the active form of vitamin D supplementation on falls. The effects of supplemental vitamin D and active forms of vitamin D were separately analyzed. A random effects model was used for the meta-analysis and risk ratios (RRs), absolute risk differences (ARDs) and 95% CI were calculated. When there was inconsistency between the RR and ARD, the results were interpreted based on the RR model, since the RR model is more consistent than the ARD model, especially for interventions designed to prevent adverse events (14, 18). We pooled the data with a random-effects model (19), and statistical heterogeneity was evaluated using the *I* ^2^ statistic. We identified additional trials that did not meet the primary analysis criteria to be included in the sensitivity analysis. STATA 16.0 (Stata Corp, College Station, TX, USA) was used to perform all meta-analyses (20). A 2-tailed *P*<0.05 was considered statistically significant.

To assess whether the relationship between vitamin D and falls was modified according to clinical features, we assessed the dose and frequency of vitamin D supplementation (≥700 IU/d; <700 IU/d); sex (only for female studies or including male and female studies); dwelling (community or institutionalized); dietary supplemental calcium; serum 25-hydroxyvitamin D concentration (≥60 or <60 nmol/L); form of vitamin D (D_3_ only or D_2_ only); the use of intermittent high doses given once a year, once every 3 or 4 months and other frequencies; and daily doses including twice a day and daily. Subgroup analysis was performed to assess whether the differences between subgroups were statistically significant.

## Results

### Studies Retrieved and Characteristics

**Figure 1:**
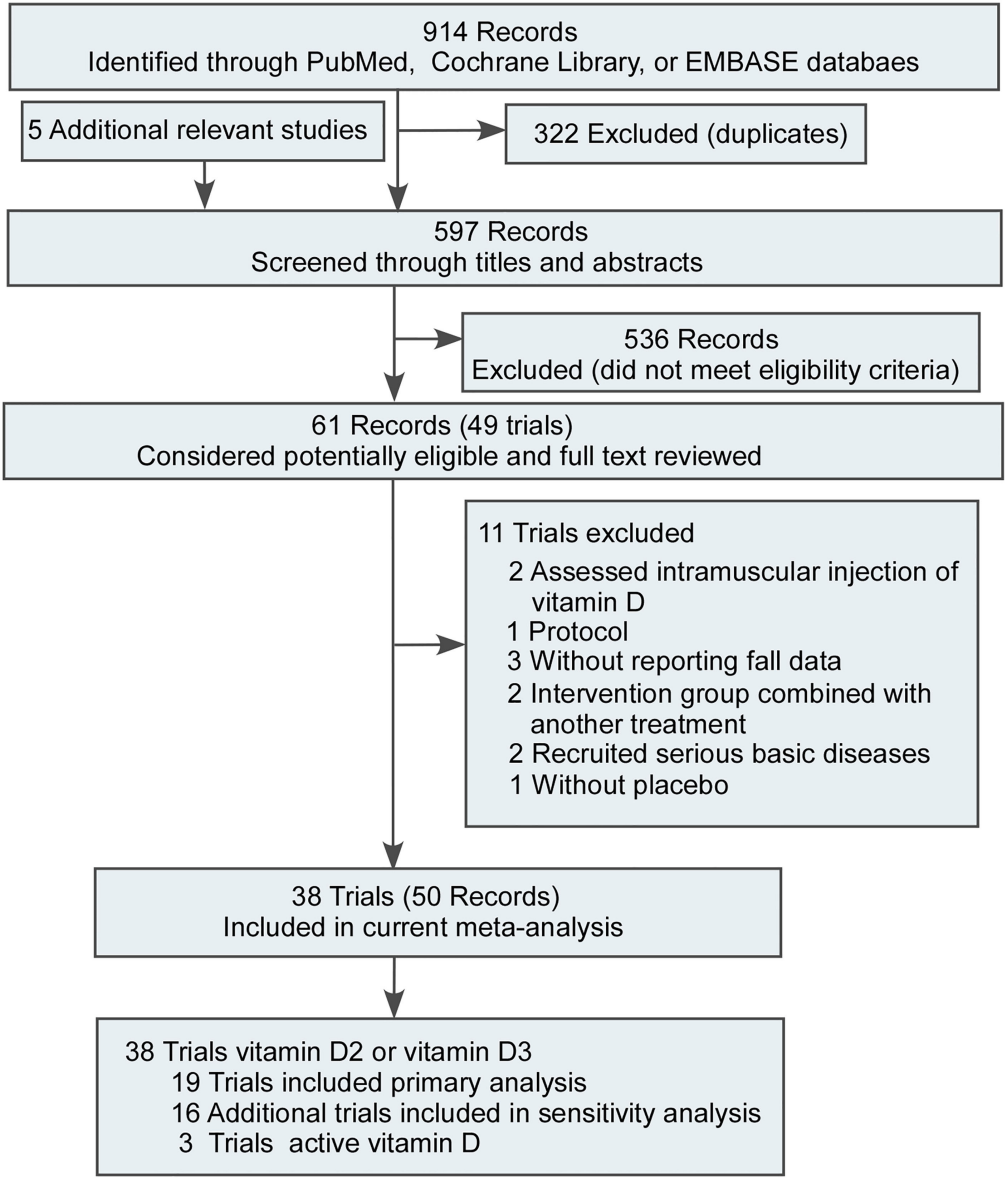
Literature Search and Screening Process.

We excluded duplicate studies and 38 RCTs (8, 21–57) including 61 350 participants in this meta-analysis (**Figure 1**). One study was shown a high risk for randomization sequence generation (46). Three studies showed a high risk in blinding of participants and personnel (44, 46, 49). One study showed a high risk in blinding of outcome assessment (44). Four studies showed a high risk in incomplete outcome data (35, 41, 44, 48). Two studies showed a high risk in selective reporting (27, 35). Most studies were of moderate or high quality (36/38). The assessment of the risk of bias were shown in **eFigures 1**, **2**. The characteristics of the included RCTs were reported in **Table 1**. Eighteen RCTs on supplemental vitamin D were identified that met our inclusion criteria for the main analysis. There were explicit fall ascertainments in trials. A previous study found that there was a difference in the rate of falling between the high-dose group and the low-dose group (2), so we divided trials into high-dose and low-dose groups based on a daily dose of 700 IU of vitamin D_2_ or D_3_.

**Figure 2:**
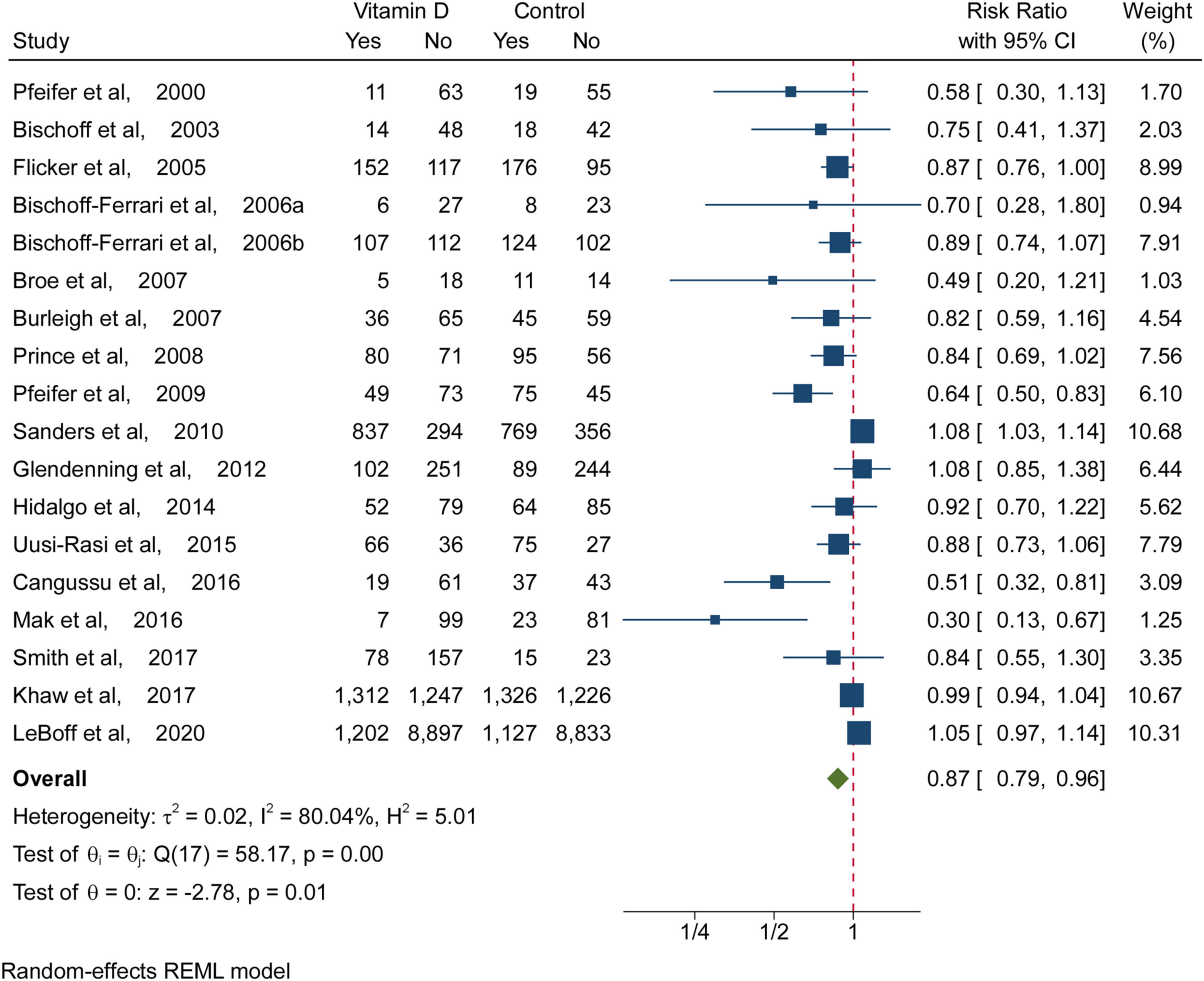
Meta-analysis Results of Vitamin D Supplementation for the Incidence of Fall. Risk ratios and 95% CIs were calculated using a random-effects model to pool data. Boxes represent relative risks, and the size of the boxes is proportional to the size of the high dose supplemental vitamin D trials included in the primary analysis. Error bars represent 95% confidence intervals.

**Table 1:** Characteristics of the included trials and participants.

Source	Study Country	Treatment	Numbe of Participants	Age(Mean ± SD)	Gender (M/F)	Dwelling	StudyLength	Change in 25-Hydroxyvitamin D Level in Intervention Group, Mean (SD), nmol/L	Osteoporotic
**Pfeifer, (** 8 **)**	Germany	800 IU Cholecalciferol + 1200 mg of calcium	148	74.7 (0.5)	0/74	Ambulatory	2 months + 1 year	25.7 (20.9) to 40.5 (27.0)	NA
		Placebo+1200 mg of calcium		74.8 (0.5)	0/74			24.6 (12.1) to 42.9 (33.1)	
**Graafmans, (** 21 **)**	The Netherlands	400 IU Cholecalciferol + estimated calcium intake from dairy products 800-1000 mg/d	354	>70	52/302	Ambulatory in homes for older individuals	7 months	Not stated	NA
		Placebo							
**Bischoff, (** 22 **)**	Switzerland	800 IU Cholecalciferol + 1200 mg calcium	122	84.9 ± 7.7	0/62	hospitalized	12 weeks	30.8 (23-55) to 65.5 (49.8-82.8)	NA
		1200 mg calcium		85.4 ± 5.9	0/60			29 (23-55) to28.5 (24.5-41.5)	
**Flicker, (** 23 **)**	Australia	600 mg of elemental calcium daily + 10,000 IU ergocalciferol once per week/1,000 IU ergocalciferol once daily	625	83.6 ± 7.8	16/297	Nursing home + Hostel	2 years	25-60 at baseline	NA
		Placebo		83.3 ± 8.8	16/296			25-60 at baseline	
**Bischoff-Ferrari, (** 57 **)**	USA	600 mg of calcium carbonate + 400 IU of cholecalciferol twice a day	89	85.6 ± 6.4	0/33	hospitalized	12 weeks	Not stated	NA
		600 mg of calcium carbonate twice a day		85.7 ± 5.9	0/31				
**Bischoff-Ferrari, (** 24 **)**	USA	700 IU of cholecalciferol + 500 mg of calcium citrate malate per day	445	71 ± 5	98/121	Ambulatory	3 years	76 (35) to 107 (38)	NA
		Placebo			101/125			73 (32) to 72 (30)	
**Broe, (** 25 **)**	USA	200 IU vitamin D daily	124	92 ± 6	7/19	Nursing home patients	5 months	45 (23) to 60 (20)	NA
		400 IU vitamin D daily		88 ± 5	7/18			53 (28) to 55 (22)	
		600 IU vitamin D daily		89 ± 6	8/17			40 (19) to 60 (20)	
		800 IU vitamin D daily		89 ± 5	7/16			54 (23) to 75 (15)	
		Placebo		86 ± 7	5/20			50 (23) to 61 (34)	
**Burleigh, (** 26 **)**	UK	cholecalciferol 800 IU + calcium 1,200 mg daily	205	82.3 ± 7.6	40/61	Geriatric medical unit	1 month	25 to 27	NA
		Calcium 1,200 mg daily		83.7 ± 7.6	44/60			22 to 22	
**Pfeifer, (** 28 **)**	Germany, Austria	800 IU vitamin D3 + 1000 mg calcium/d	242	77 ± 4	30/91	Ambulatory individuals	20 months	55.4 (18.5) to 84.5 (18.0)	NA
		Placebo + 1000 mg calcium		76 ± 4	31/90			53.8 (18.4) to 56.6 (20)	
**Prince, (** 27 **)**	Australia	Ergocalciferol, 1000 IU/d + calcium citrate, 1000 mg/d	302	77.0 ± 4.2	0/151	Community dwelling	1 year	45 to 60	None
		Placebo + calcium citrate, 1000 mg/d		77.4 ± 5.0	0/151			44.3 to 49	
**Sanders, (** 29 **)**	Australia	A single oral dose of cholecalciferol 500 000 IU in autumn or winter	2,256	76	0/1131	Community dwelling	3 to 5 years	Not stated	Osteoporosis diagnosis 1.0% (n = 23/2256)
		Placebo		76.1	0/1125				
**Glendenning, (** 30 **)**	Australia	Vitamin D3 150,000 IU every 3 months	686	76.9 ± 4.0	0/353	Community dwelling	9 months	65.0 (17.8) to 74.6 (25.8)	NA
		Placebo		76.5 ± 4.0	0/333			66.5 (27.1) to 60.2 (26.3)	
**Hidalgo, (**31)	Spain	800 IU of vitamin D3 + 1,000 mg of calcium daily	508	72.6 ± 4.9	85/103	Community dwelling	2 years	86.77 (41.0) at baseline	None
		Placebo		72.4 ± 5.2	105/105			79.3 (42.7) at baseline	
**Uusi-Rasi, (** 32 **)** ^a^	Finland	Vitamin D3 800 IU vitamin/d	409	74.1 ± 2.9	0/204	home-dwelling	2 years	63 to 93	NA
		Placebo		74.3 ± 3.0	0/205			69 to 69	
**Cangussu, (** 33 **)**	Brazil	vitamin D3 1,000 IU/day/orally	160	58.8 ± 6.6	0/80	Ambulatory	9 months	37.29 to 68.37	None
		Placebo		59.3 ± 6.7	0/80			42.0 to 34.3	
**Mak, (** 34 **)**	Australia	250,000 IU vitamin D (loading dose)+800 IU vitamin D and 500 mg calcium daily	218	83.7 ± 7.5	27/84	Community dwelling	4 weeks	55.6 to 77	NA
		placebo+800 IU vitamin D and 500 mg calcium daily		84.1 ± 7.0	23/84			49.6 to 74	
**Smith, (** 35 **)**	USA	400 IU vitamin D3 daily	273	66	0/67	Community dwelling	12 months	36 at baseline	NA
		800-4800 IU vitamin D3 daily			0/168				
		Placebo			0/38				
**Khaw, (** 36 **)**	New Zealand	200 000 IU followed by 100 000 IU monthly	5108	65.9 ± 8.3	1512/1046	Ambulatory	3.4 years	63 (24) at baseline	Osteoporosis diagnosis
		Placebo			1457/1093				1.4%(N=71/5108)
**LeBoff, (** 37 **)**	USA	2000 IU/day of vitamin D3	25,871	67.13 (7.05)	6380/6547	Ambulatory	5.3 years	76.8 (25) at baseline	NA
		Placebo		67.14 (7.08)	6406/6538			76.6 (25) at baseline	

a We extracted only the information and data in placebo without exercise and vitamin D (800 IU/d) without exercise groups. NA, not available.

### Vitamin D and Fall Risk

**Figure 2** shows the comparison of vitamin D with placebo or no treatment. Compared with a placebo or no treatment, vitamin D (≥700 IU/d) prevented falling (RR, 0.87 [95% CI 0.79 to 0.96]; ARD, -0.06 [95% CI, -0.10 to -0.02], **Figure 2** and **eFigure 3**). The results suggested that daily intake of high doses of vitamin D reduced the risk of falls in older individuals by 13%, and the number needed to treat was 17 (95% CI, 10 to 50). However, there was no significant association of low-dose vitamin D with falling (RR, 1.09 [95% CI, 0.90 to 1.32]; ARD, 0.03 [95% CI, -0.05 to 0.12], **eFigures 4, 5**). **eFigure 6** in the Supplement, a contour-enhanced funnel plot, did reveal significant publication bias.

### Primary Subgroup Analyses

**Figure 3:**
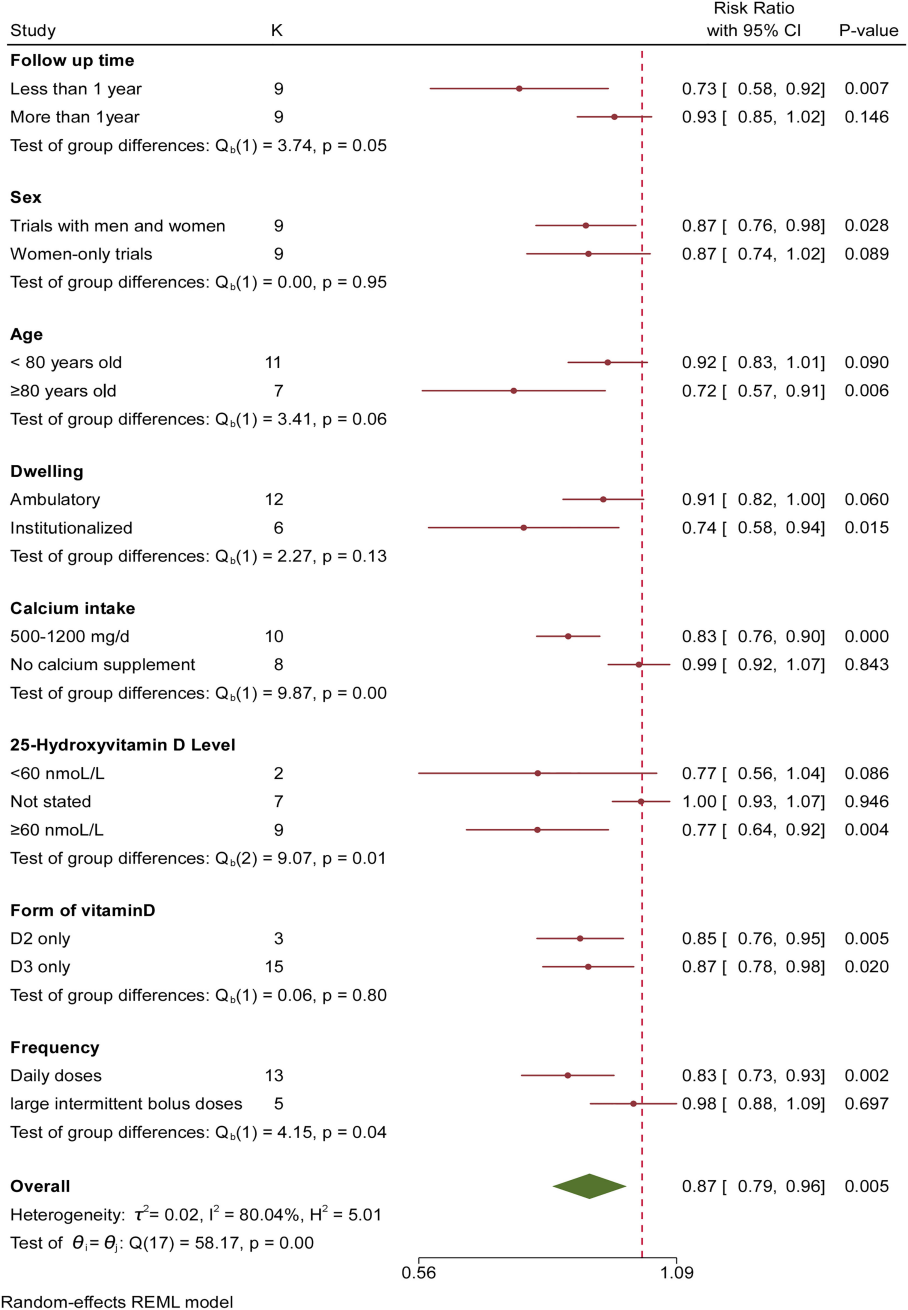
Subgroup Analysis of Association Between Vitamin D Supplementation and Fall Incidence for Each Variable. Risk ratios and 95% CIs were calculated using a random-effects model to pool data. Boxes represent relative risks, and the size of the boxes is proportional to the size of the high dose supplemental vitamin D trials included in the primary analysis. Error bars represent 95% confidence intervals.

As a result of statistical heterogeneity, we performed a subgroup analysis for high doses of supplemental vitamin D (more than 700 IU). The role of vitamin D was highly regulated by treatment duration: fall reduction was 27% with less than 12 months of treatment (RR, 0.73 [95% CI, 0.58 to 0.92]) compared with 7% with 12 months or more of treatment (RR, 0.93 [95% CI, 0.85 to 1.02], **Figure 3**). There was no difference in the number of falls between women-only trials and trials with men and women (*P*=0.95). The pooled risk reduction for falling was 28% in trials in which participants were older than 80 years old (RR, 0.72 [95% CI, 0.57 to 0.91]) compared with 8% for trials in which participants were less than 80 years old (RR, 0.92 [95% CI, 0.83 to 1.01]). Therefore, participants older than 80 years old benefited more from supplemental vitamin D. Vitamin D was equally effective for elderly individuals in community (RR, 0.91 [95% CI, 0.82 to 1.00]) and institutionalized dwellings (RR, 0.74 [95% CI, 0.58 to 0.94]). Vitamin D_2_ and vitamin D_3_ achieved similar effects (*P*=0.80). The role of vitamin D was highly modulated by supplemental calcium: no calcium supplement did not reduce the risk of falls (RR, 0.99 [95% CI, 0.92 to 1.07]). However, the pooled risk reduction for falling was 17% (RR, 0.83 [95% CI, 0.76 to 0.90]) in trials with supplemental calcium of 500-1200 mg/d. The results implied that the efficacy of vitamin D depended on additional calcium supplementation. The pooled risk reduction for falling was 23% in trials with 25(OH)D concentrations ≥60 nmol/l (RR, 0.77 [95% CI, 0.64 to 0.92]) compared with trials with 25(OH)D concentrations <60 nmol/l (RR, 0.77 [95% CI, 0.56 to 1.04]). The results suggested that a 25(OH)D concentration of 60 nmol/l was important for preventing falls. In addition, the pooled risk reduction for falling was 17% in trials with high daily doses (RR, 0.83 [95% CI, 0.73 to 0.93]) compared with trials with large intermittent bolus doses (RR, 0.98 [95% CI, 0.88 to 1.09]). The results suggested that high-dose bolus vitamin D supplementation did not prevent falls.

### Sensitivity Analysis of Supplemental Vitamin D

To understand the reliability and accuracy of the results, we performed sensitivity analysis. We included the studies eliminated in the primary analysis in sensitivity analysis. Twelve eliminated studies were excluded for unclear definitions of falling (38–41, 43, 45, 47, 49–51, 53). These trial designs were not double-blind, or they did not describe the generation of random sequences (41, 42, 44–46, 48–51). Sixteen additional RCTs were included to examine the effect, which expanded the participant population to 55 318. The characteristics of these studies are shown in **Table 2**. The results showed that compared with a placebo or no treatment, vitamin D prevented falling (RR, 0.96 [95% CI, 0.92 to 1.00]; ARD, -0.03 [95% CI, -0.05 to -0.01], **Table 3** and **eFigures 7, 8**), which was consistent with the primary analysis. The number of effects was reduced by these additional studies, but the benefits remained statistically significant.

**Table 2:** Trials of supplemental vitamin D excluded from the primary analyses but included in sensitivity analyses.

Source	Study Country	Treatment	Numbe of Participants	Age(Mean ± SD)	Gender (M/F)	Dwelling	StudyLength	Change in 25-Hydroxyvitamin D Level in Intervention Group, Mean (SD), nmol/L	Osteoporotic
**Chapuy, (** 38 **)**	France	800 IU Cholecalciferol + 1200 mg/d of calcium	583	85 (7)	0/393	Ambulatory in homes for the elderly	2 years	21.3 (13.3) to 77.5	None
		Placebo			0/190			22.8 (17.3) to 15	
**Trivedi, (** 39 **)**	UK	800 IU vitamin D3 (100 000 IU every 4 months)	2386	74.8 (4.6)	1019/326	Community dwelling	1 year	74.3 (20.7) at 48 months	NA
		Placebo		74.7 (4.6)	1018/323			53.4 (21.1) at 48 months	
**Latham, (** 40 **)**	New Zealand, Australia	300 000 IU Cholecalciferol once + no calcium	243	79 (77–80)	57/64	Acute care recruitment of frail elderly	6 months	37.5 (35-45) to 60	NA
		Placebo		80 (78–81)	57/65			47.5 (40-52.5) to 47.5	
**Harwood, (** 41 **)**	UK	800 IU vitamin D3 +1g calcium	150	81 (67-92)	0/113	Patients in rehabilitationwards, previously community dwelling	1 year	30 (6-75) to 50	None
		Placebo			0/37			30 (12-64) to 27	
**Larsen, (** 42 **)**	Denmark	1000 mg Ca+400 IU vitamin D3/Day	4256	74 (65–103)	843/1273	Community dwelling	42 months	Not stated	NA
		Control			1974/2983				
**Grant, (** 43 **)**	UK	800 IU vitamin D3 with or without 1000 mg calcium per day	5292	77 ± 6	409/2240	Individuals who were mobile before developing a low trauma fracture	2 years	38 (16) to 62 (19.5)	NA
		Placebo			422/2241			38 (16) to 45.8 (18)	
**Porthouse, (** 44 **)**	UK	Vitamin D3 800 IU + 1000 mg calcium	2541	77.0 ± 5.10	0/914	community-dwelling	1 year	Not stated	NA
		No supplementation		76.7 ± 5.02	0/1627				
**Law, (** 45 **)** ^a^	UK	1100 IU vitamin D2 (100 000 IU ergocalciferol every 3 months)	3137	85	929/2788	Patients living in residential	10 months	47 (35-102) to 74 (52-110)	NA
		No treatment (no placebo)						Not stated	
**Kärkkäinen, (** 46 **)**	Finland	800 IU vitamin D3 + 1g calcium	3432	67.4 ± 1.9	0/1718	Community dwelling	3 years	Not stated	NA
		Control group (no placebo)		67.3 ± 1.8	0/1714				
**Wood, (** 47 **)**	UK	1100 IU vitamin D3 daily	305	60–70	0/203	Community	12 months	33 to 70	NA
		Placebo			0/102			36 to 32	
**Rizzoli, (** 48 **)**	13 countries	1000 IU vitamin D3 + 1g calcium	518	66.9 ± 8.3	41/372	Ambulatory	6 months	44.0 (14.9) to 67	Yes
		Control		66.6 ± 8.0	8/97			44.4 (13.3) to 45	
**Houston, (** 49 **)** ^a^	USA RCT(Cluster)	Vitamin D3 two 50,000 IU capsules/month;	68	77.6 ± 9.0	8/30	Community dwelling	5 months	22.5 (12.2) at baseline	NA
		Placebo (400 IU vitamin E/month)		78.2 ± 8.4	11/19			18.9 (10.6) at baseline	
**Hansen, (** 50 **)**	USA	800 IU vitamin D3 daily or twice monthly 50,000 IU vitamin D3	230	61	0/154	Community dwelling	12 months	53 to 86	None
		Placebo			0/76			53 to 45	
**Levis, (** 51 **)**	USA	4,000 IU cholecalciferol daily	130	71.8 ± 6.3	66/0	Ambulatory	9 months	58 to 115	NA
		Placebo		73.0 ± 7.3	64/0			57 to 60	
**Hin, (** 53 **)**	UK	2000 IU/day	305	71 ± 6	52/50	Community-dwelling	1 year	Not stated	NA
		4000 IU/day		72 ± 6	51/51				
		Placebo		72 ± 6	52/49				
**Dhaliwal, (** 52 **)**	USA	2400, 3600 or 4800IU vitamin D3 +1200 mg calcium daily	260	67.8	0/130	Community-dwelling	3 years	94 achieved	None
		Placebo +1200 mg calcium daily		69.0	0/130			52 achieved	

a They is a randomized controlled trial of cluster design. They was adjusted for the number of participants. NA, not available.

**Table 3:** Sensitivity analysis of the eighteen trials from the primary analysis and the sixteen eligible trials that did not meet the criteria for the primary analysis.

Study	Number of participants	Vitamin D		Placebo		Fall, RR (95% CI)
		With Fall	Total	With Fall	Total	
Pooled primary analysis of the eighteen trials	31355	4135	15850	4096	15505	0.87 (0.79-0.96)
Heterogeneity: τ^2 =^ 0.02; I^2 =^ 80.04%; H^2 =^ 5.01 Test of θ=0: z =-2.78 (*P* =0.01)						
Sensitivity analysis including the sixteen trials that did not meet criteria for primary analysis						
Trivedi, (39)	2038	254	1027	261	1011	0.96 (0.83-1.11)
Latham, (40)	222	64	108	60	114	1.13 (0.89-1.42)
Chapuy, (38)	583	251	393	118	190	1.03 (0.90-1.18)
Harwood, (41)	119	15	84	13	35	0.48 (0.26-0.90)
Larsen, (42)	4607	466	2491	403	2116	0.98 (0.87-1.11)
Grant, (43)	5292	380	2649	381	2643	1.00 (0.87-1.13)
Porthouse, (44)	2541	289	914	498	1627	1.03 (0.92-1.16)
Law, (45)	3137	770	1762	833	1955	1.03 (0.95-1.10)
Kärkkäinen, (46)	3139	812	1566	833	1573	0.98 (0.92-1.05)
Wood, (47)	196	27	96	31	100	0.91 (0.59-1.40)
Rizzoli, (48)	518	65	413	21	105	0.79 (0.51-1.23)
Houston, (49)	66	11	37	12	29	0.72 (0.37-1.39)
Hansen, (50)	230	46	154	23	76	0.99 (0.65-1.50)
Hin, (53)	305	34	204	14	101	1.20 (0.68-2.14)
Dhaliwal, (52)	260	51	130	50	130	1.02 (0.75-1.38)
Levis, (51)	130	8	66	11	64	0.71 (0.30-1.64)
Pooled sensitivity analysis	55318	7678	27944	7658	27374	0.96 (0.92-1.00)
Heterogeneity: τ^2 =^ 0.00; I^2 =^ 47.98%; H^2 =^ 1.92 Test of θ=0: z =-1.98 (*P* =0.05)						

I^2^ estimates above 25% are considered to represent modest heterogeneity, and values above 50% represent large heterogeneity beyond chance.

### Active Vitamin D Supplementation and Fall Risk

Three RCTs (54–56) on the active forms of vitamin D met our inclusion criteria (**eTable 2**). There were clear definitions of falling in these trials. However, the random sequence generation was not described in one trial (56), so we excluded it from the primary analysis. This study was included in the sensitivity analysis. Compared with a placebo or no treatment, active forms of vitamin D prevented falls (RR, 0.78 [95% CI, 0.64 to 0.95]; ARD, -0.09 [95% CI, -0.20 to 0.02], **eFigures 9, 10**). Active vitamin D intake can reduce the risk of falls by 22%, based on the RR. The sensitivity analysis was consistent with the primary analysis (**eFigure 11**).

## Discussion

This meta-analysis included thirty-eight double-blind RCTs with 61 350 elderly individuals treated with vitamin D for 2 to 63 months. Seventeen RCTs were excluded from all primary analyses because they did not meet the criteria. The pooled ARD in the primary analysis indicated that 17 people need vitamin D treatment to prevent one person from falling and daily intake of high doses of vitamin D reduced the risk of falls in elderly individuals by 13%. When 16 additional RCTs were included in the sensitivity analysis, these results were not modulated. However, the effectiveness of vitamin D for preventing falling depended on the dose, time, supplemental calcium, 25-hydroxyvitamin D level and frequency, according to the subgroup analysis.

Not only can a fall cause serious injury or death but elderly people who have experienced a fall also have increased anxiety and depression (58, 59), and their quality of life is reduced (60). However, there is still much controversy about the role of vitamin D in preventing falls. Therefore, we conducted this study to evaluate the effectiveness of vitamin D in preventing falls. A meta-analysis conducted by Bischoff-Ferrari et al. showed that vitamin D reduced the risk of falls among healthy ambulatory or institutionalized older individuals by 22% (13). However, they included a cluster experiment with a large sample (45) did not adjust for the number of participants. There was no distinction between the form and dose of vitamin D in their study. This meta-analysis did not find a significant association between low vitamin D intake and fall prevention. (RR, 1.09 [95% CI, 0.90 to 1.32]; ARD, 0.03 [95% CI, -0.05 to 0.12]). The results manifested that the efficacy has nothing to do with the form of vitamin D (vitamin D_2_, D_3_ and active forms of vitamin D) in preventing falls. In a meta-analysis from 2009 (2), it was reported that Vitamin D has nothing to do with calcium intake. However, they did not compare vitamin D combined with calcium supplementation with vitamin D alone. We found that supplemental calcium influenced the effect of vitamin D on the prevention of falls in the subgroup analysis. Therefore, we suggest that Vitamin D and calcium should be supplemented at the same time. In less than 1 year of treatment, the risk of taking high-dose vitamin D was reduced by 27% and a sustained 7% fall reduction for 1-5.3 years. These results were consistent with those of a previous study (2).

A Cochrane review suggested that vitamin D did not appear to reduce falls (61). This difference might be because they did not include some high-quality RCTs. It has been found that vitamin D supplementation did not prevent falls in a prior study, and there was no difference between high-dose and low-dose vitamin D. The possible reason for the differences was that Bolland et al. excluded a large amount of literature on vitamin D from their meta-analysis. Their reason was that calcium supplements have uncommon but clinically important side effects (62). However, a recent meta-analysis conducted by Chung reported that supplemental calcium within tolerable upper intake levels (2000 to 2500 mg/d), healthy adults were generally not associated with a risk of cardiovascular disease (63). We believe that when analyzing the role of vitamin D, some studies could not be excluded despite the side effects of calcium, which would lead to unreliable results. Current research showed that vitamin D and calcium can reduce the risk of falls by 18%. Guirguis-Blake performed random-effects meta-analyses and the conclusion was that vitamin D supplementation has mixed effects in preventing falls (10). However, they only included a small part of the research on vitamin D. Their review was focused on community-dwelling older adults. They reported that large intermittent bolus doses increased the rate of fall. A previous RCT reported that in this healthy and active adult group, high doses of vitamin D did not prevent falls or fractures (36). In this meta-analysis, it was shown that large intermittent bolus doses of vitamin D had no preventive effect on falls, which was consistent with a previous study (10, 36).

Davies reported that a 6% reduction in the risk of fall associated with vitamin D would be cost effective (64). The results reported here showed that daily intake of high doses of vitamin D could reduce the risk of falling in elderly individuals by 13%, which was higher than 6%. Therefore, vitamin D supplementation was cost effective.

## Limitations

This study had several limitations. First, the results of some meta-analysis were moderately heterogeneous because several studies reported negative results regarding high-dose bolus vitamin D. High-dose bolus vitamin D was proven to be useless in fall prevention in some RCTs (29, 30, 34, 36). Second, some small sample studies might affect the results. Then, the results showed the relationship between 25(OH) D concentration and falls. However, there was no RCTs to confirm the relationship between 25(OH) D concentration and falls. In this regard, further research is needed to determine the relationship between 25(OH) D concentration and falls. In addition, a publication bias has likely affected the results presented in this review.

## Conclusions

In this study, doses of 700 IU to 2000 IU of supplemental vitamin D per day were associated with a lower risk of falling among ambulatory and institutionalized older adults. This benefit might depend on additional calcium supplementation. However, this conclusion should be cautiously interpreted, given the small differences in outcomes.

## Data Availability Statement

The original contributions presented in the study are included in the article/**Supplementary Material**. Further inquiries can be directed to the corresponding authors.

## Author Contributions

Conception and design, F-LW, C-PZ, WW, and J-XQ; Analysis and interpretation of the data, F-LW, TL, Q-YG, YH, C-PZ, WW, and J-XQ; Drafting of the article, F-LW; Critical revision of the article for important intellectual content, Q-YG, YH, C-PZ, WW, and J-XQ; Final approval of the article, F-LW, TL, Q-YG, YH, C-PZ, WW, and J-XQ; Statistical expertise, F-LW, TL, and YH; Obtaining of funding, J-XQ; Administrative, technical, or logistic support, YH, C-PZ, WW, and J-XQ; Collection and assembly of data, F-LW, TL, YH, C-PZ, WW, and J-XQ; All authors contributed to the article and approved the submitted version.

## Funding

This study was supported by the National Natural Science Foundation of China (No. 81871818), Tangdu Hospital Seed Talent Program (F-LW) and Social Talent Fund of Tangdu Hospital (No.2021SHRC034). The funding body had no role in the design of the study, data collection, analysis, interpretation or in writing the manuscript.

## Conflict of Interest

The authors declare that the research was conducted in the absence of any commercial or financial relationships that could be construed as a potential conflict of interest.

## Publisher’s Note

All claims expressed in this article are solely those of the authors and do not necessarily represent those of their affiliated organizations, or those of the publisher, the editors and the reviewers. Any product that may be evaluated in this article, or claim that may be made by its manufacturer, is not guaranteed or endorsed by the publisher.
