# Supplementary material for: Association Between Vitamin D Supplementation and Fall Prevention
Source: Front Endocrinol (Lausanne). 2022 Aug 10;13:919839. doi: 10.3389/fendo.2022.919839 (PMC9399608; doi:10.3389/fendo.2022.919839)
Supplement: Supplementary file 1 [file DataSheet_1.docx]

**Supplement materials**

**1. Figures** (eFigure 1- eFigure 11)

**eFigure 1.** Number/Proportions of trials that met each criterion for risk of bias across the 38 included trials.

**eFigure 2.** Results of the risk of bias for 38 included trials.

**eFigure** **3**. Meta-analysis results of high dose (≥700UI) vitamin D supplementation for the incidence of fall (ARD).

**eFigure 4.** Meta-analysis results of low dose (＜700UI) vitamin D supplementation for the incidence of fall (RR).

**eFigure 5.** Meta-analysis results of low dose (＜700UI) vitamin D supplementation for the incidence of fall (ARD).

**eFigure 6.** Contour-enhanced funnel plot of association between vitamin D supplementation and fall incidence.

**eFigure 7.** Sensitivity analysis of the eighteen trials from the primary analysis and the seventeen eligible trials that did not meet the criteria for the primary analysis (RR).

**eFigure 8.** Sensitivity analysis of the eighteen trials from the primary analysis and the seventeen eligible trials that did not meet the criteria for the primary analysis (ARD).

**eFigure 9.** Randomized controlled trials of active forms of vitamin D included in the primary analysis (RR).

**eFigure 10.** Randomized controlled trials of active forms of vitamin D included in the primary analysis (ARD).

**eFigure 11.** Randomized controlled trials of active forms of Vitamin D included in sensitivity analysis.

**2. Tables** (eTable 1- eTable 2)

**eTable 1.** Search strategy for each database.

**eTable 2.** Randomised controlled trials of active forms of vitamin D included in the primary analyses and sensitivity analysis.

**eFigure 1. Number/Proportions of trials that met each criterion for risk of bias across the 38 included trials.**

**
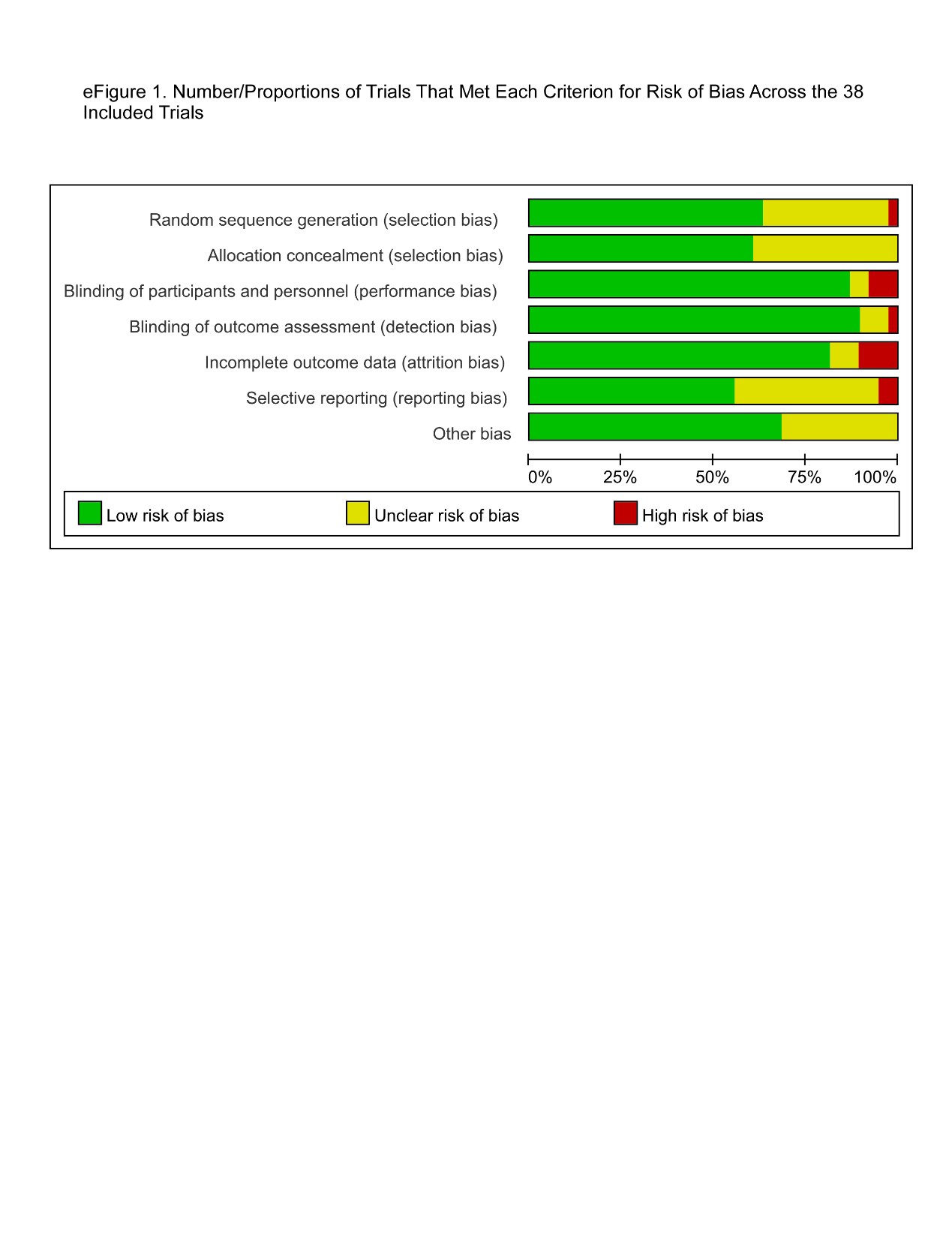
**

**eFigure 2. Results of the risk of bias for 38 included trials.**


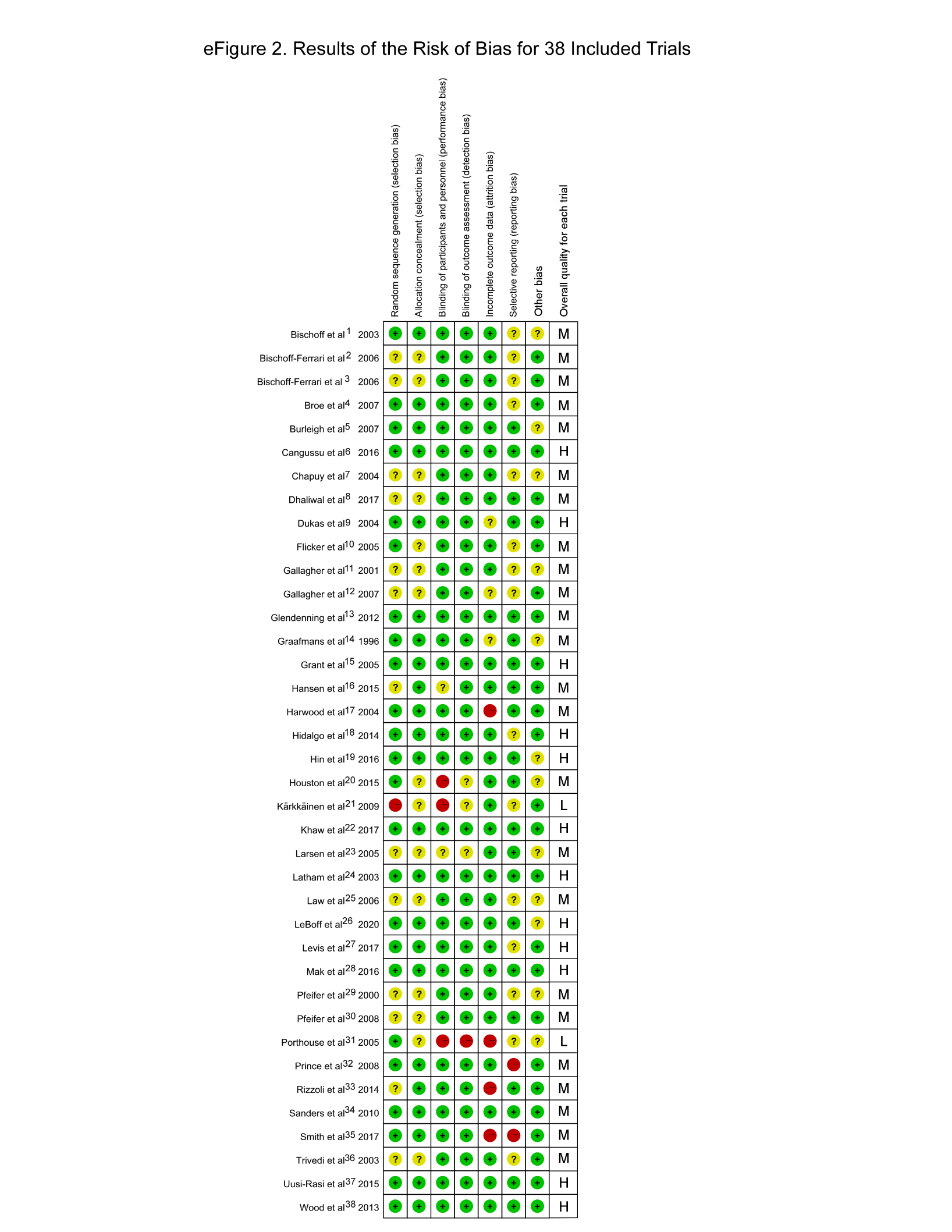


**eFigure** **3**. **Meta-analysis results of high dose (≥700UI) vitamin D supplementation for the incidence of fall (ARD).**


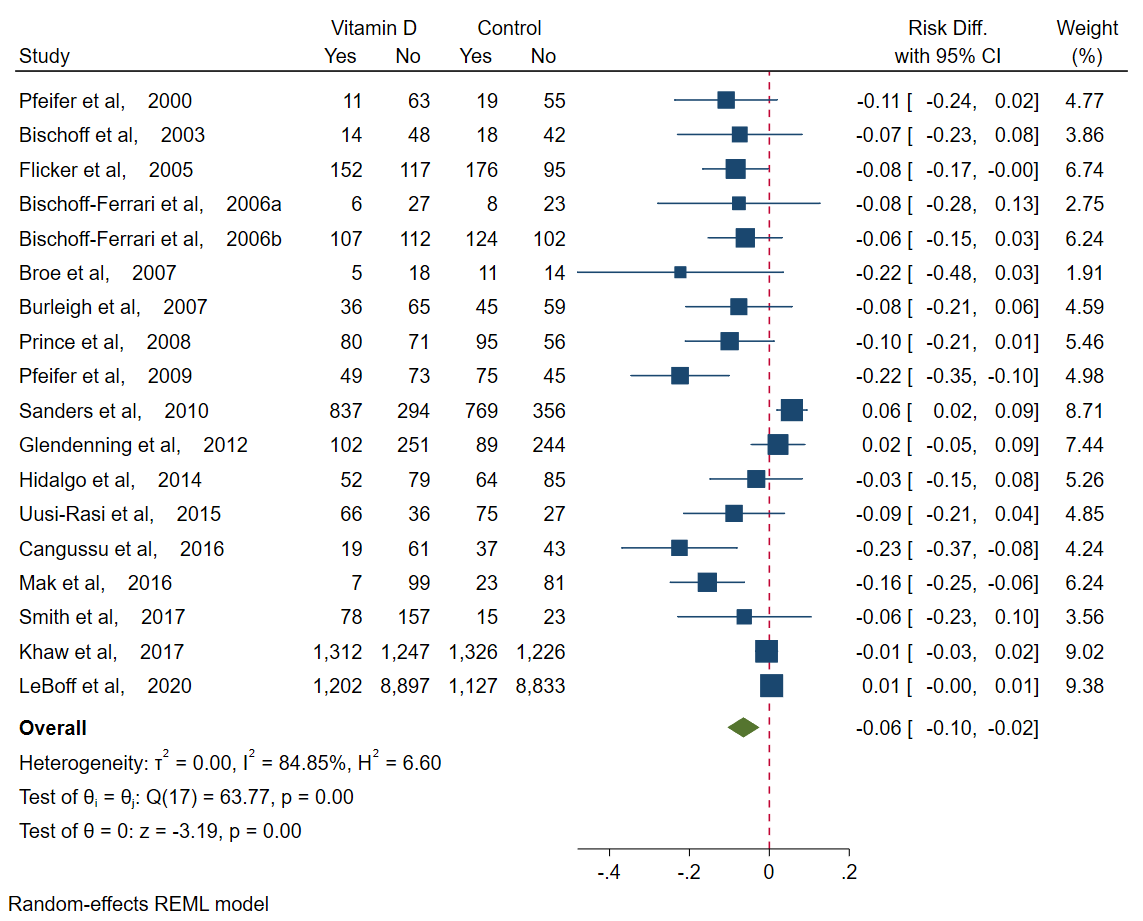


**eFigure 4.** **Meta-analysis results of low dose (＜700UI) vitamin D supplementation for the incidence of fall (RR).**


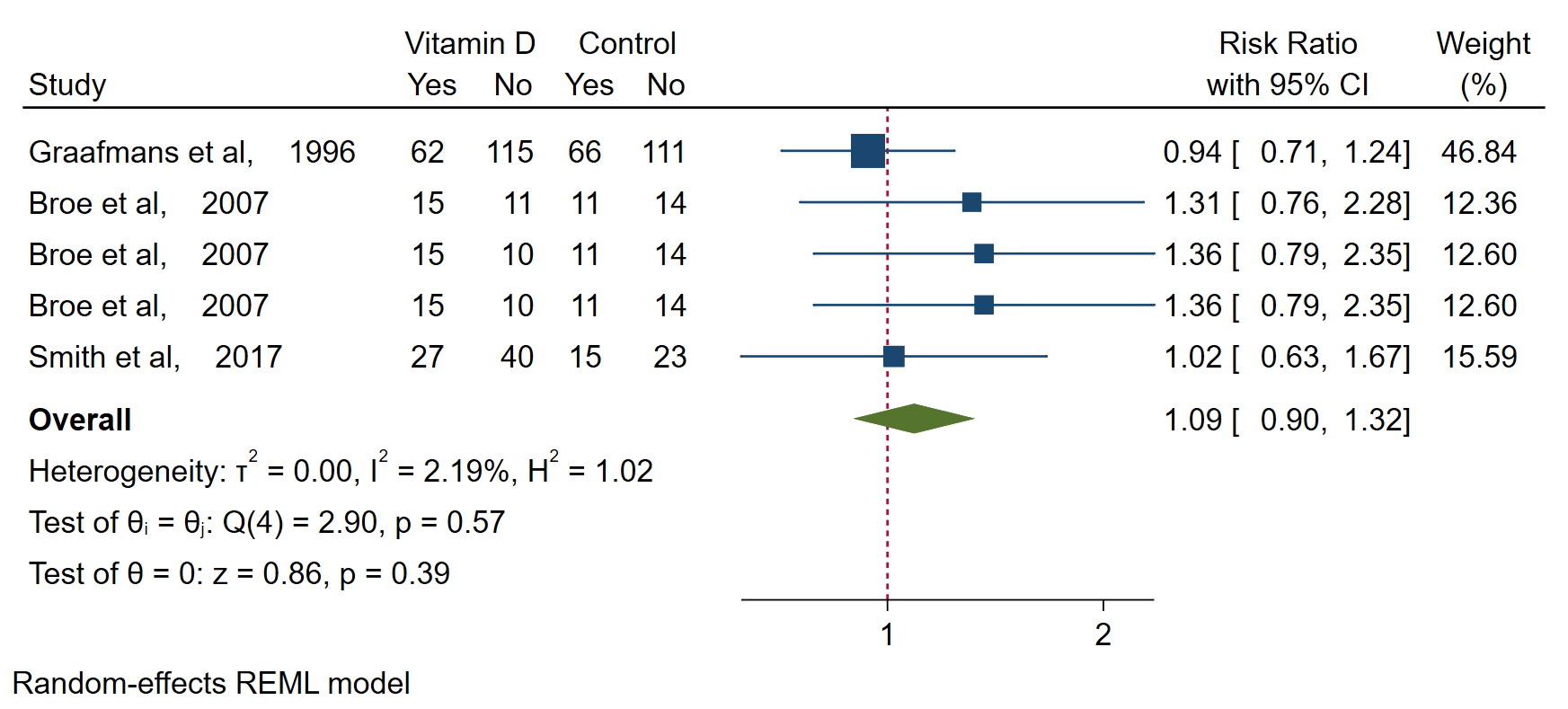


**eFigure 5.** **Meta-analysis results of low dose (＜700UI) vitamin D supplementation for the incidence of fall (ARD).**


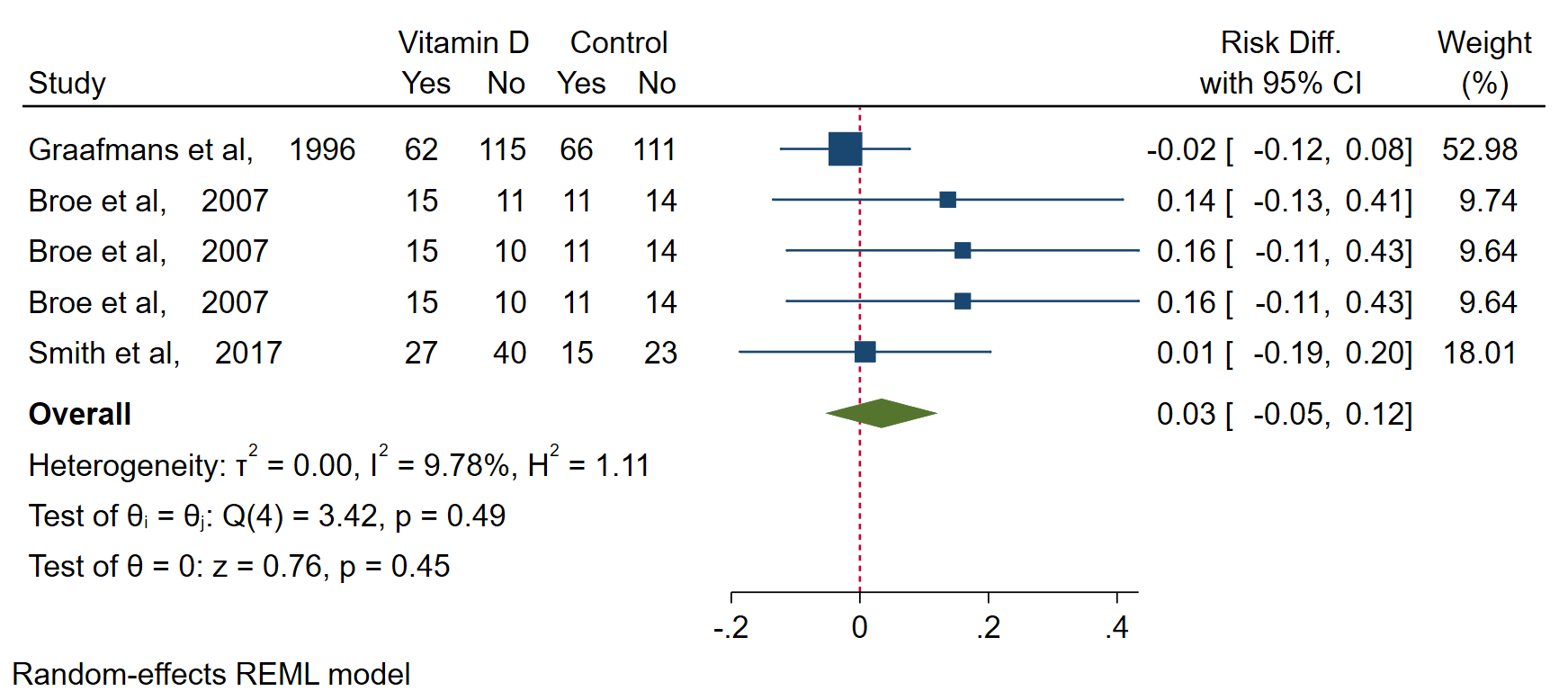


**eFigure 6.** **Contour-enhanced funnel plot of association between vitamin D supplementation and fall incidence.**

**
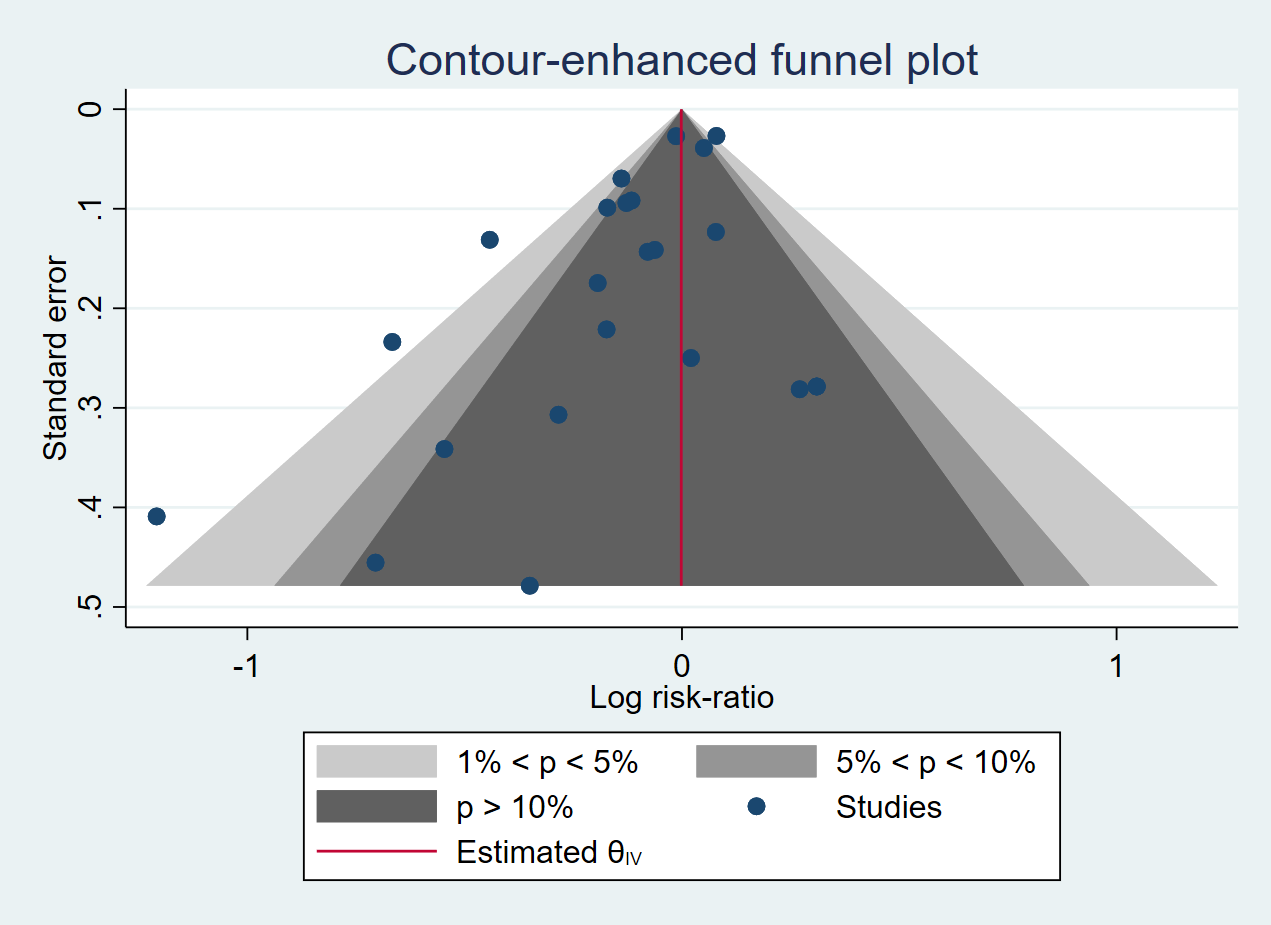
**

**eFigure 7.** **Sensitivity analysis of the eighteen trials from the primary analysis and the seventeen eligible trials that did not meet the criteria for the primary analysis.**


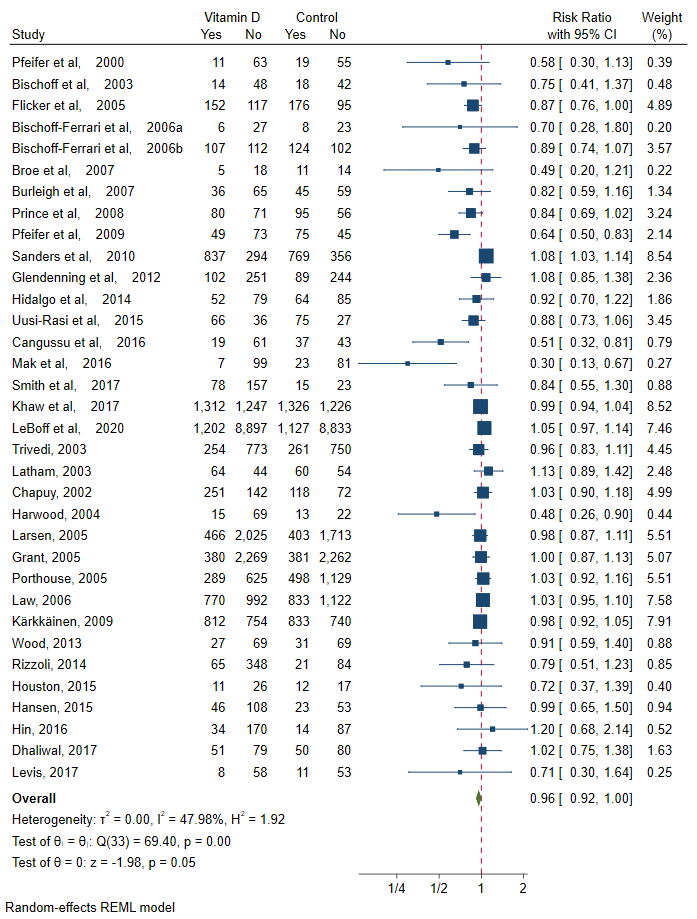


**eFigure 8.** **Sensitivity analysis of the eighteen trials from the primary analysis and the seventeen eligible trials that did not meet the criteria for the primary analysis (ARD).**


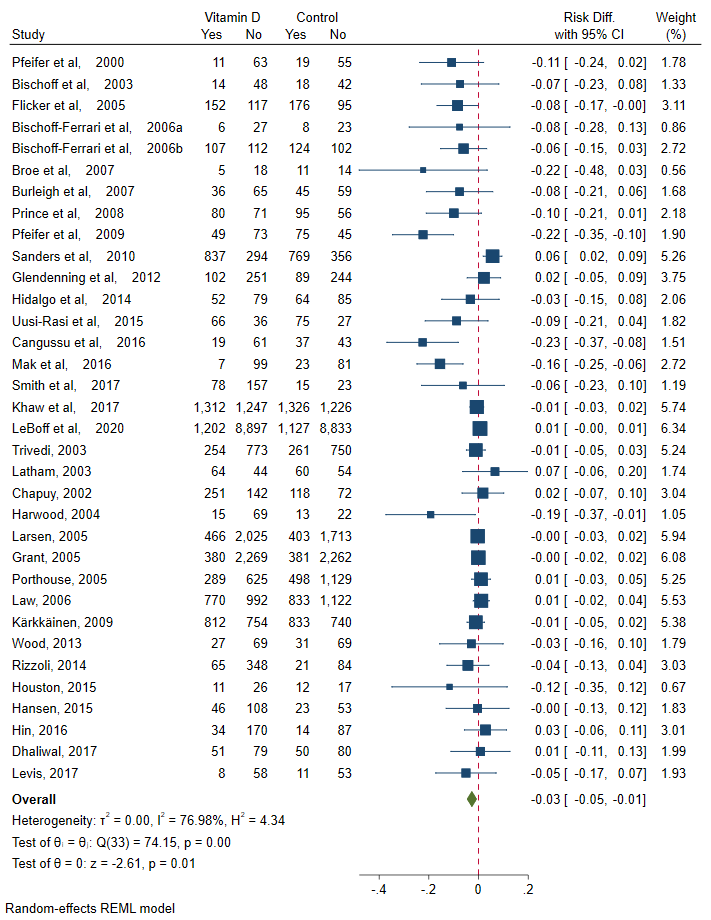


**eFigure 9. Randomized controlled trials of active forms of vitamin D included in the primary analysis (RR).**

**
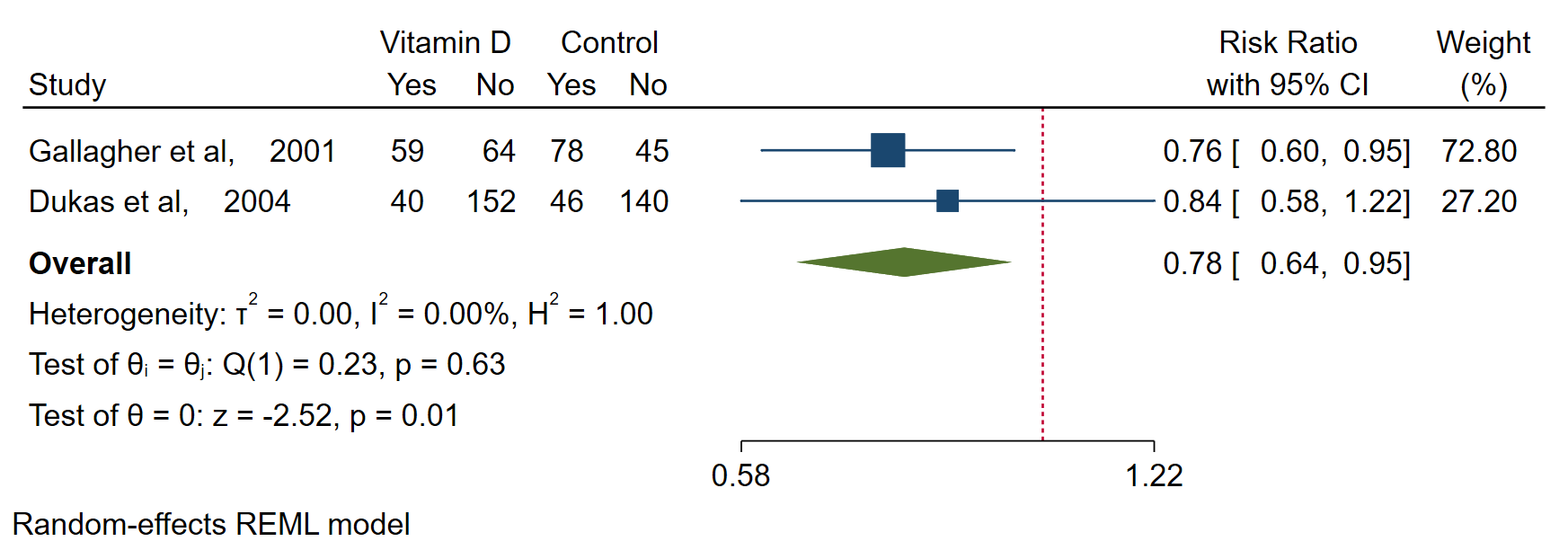
**

**eFigure 10. Randomized controlled trials of active forms of vitamin D included in the primary analysis (ARD).**

**
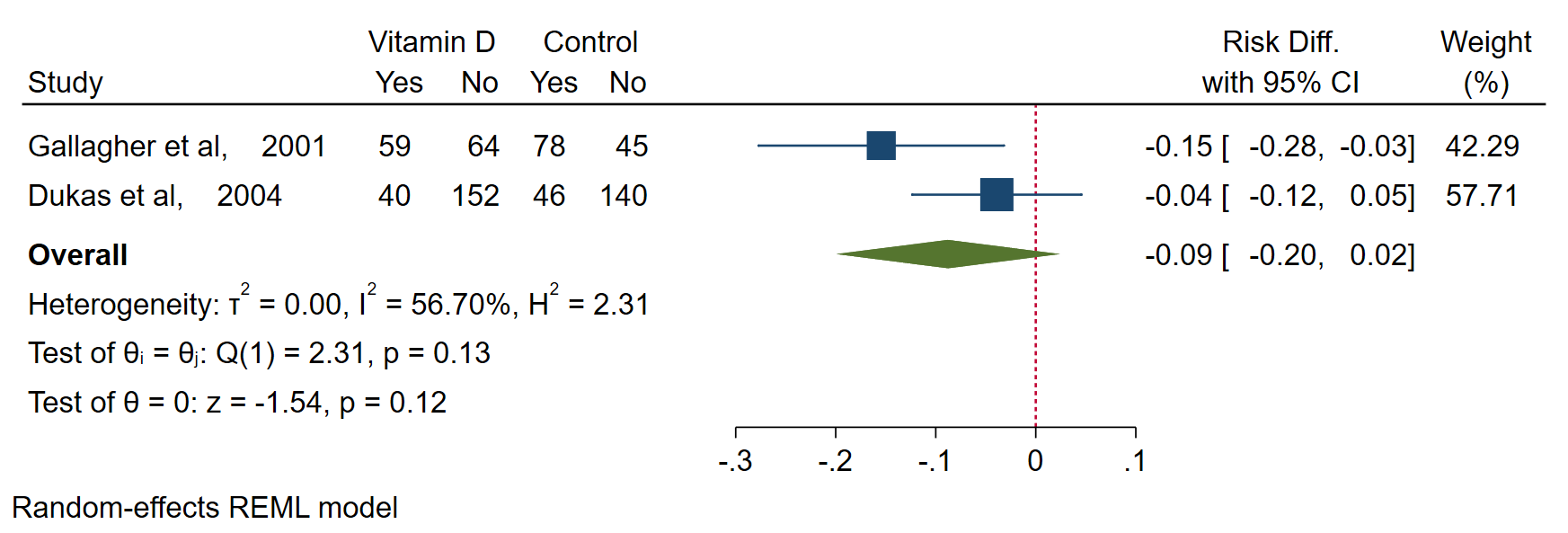
**

**eFigure 11. Randomized controlled trials of active forms of Vitamin D included in sensitivity analysis.**
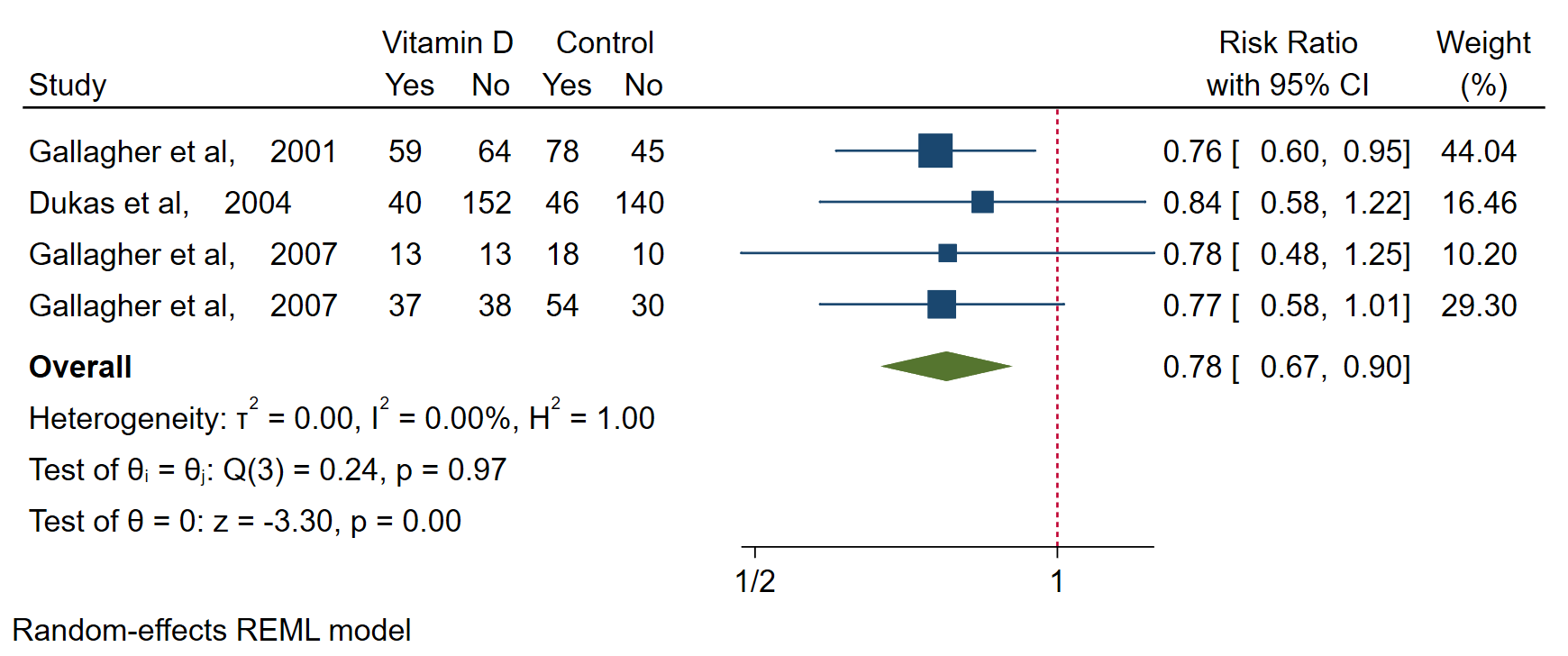


**eTable 1. Search strategy for each database.**

| Database | Search strategy |
| --- | --- |
| Pubmed | #1 "vitamin d"[MeSH Terms] OR "vitamin d"[All Fields] OR "ergocalciferols"[MeSH Terms] OR "ergocalciferols"[All Fields]  #2 ((falls[MeSH Terms]) OR (falls))  #3 (Randomized Controlled Trial [Publication Type])  #4 #1 and #2  #5 #3 and #4 |
| embase | #1 'vitamin d'/exp OR 'vitamin d'  #2 'falls'/exp OR 'falls'  #3 [ Randomized Controlled Trial]/lim  #4 #1 or #2 #5 #3 and 4 |
| Cochrane library | #1 vitamin d:ti,ab,kw (Word variations have been searched)  #2 falls:ti,ab,kw (Word variations have been searched)  #3 #1 and #2 (restricted as Trials) |

**eTable 2. Randomized controlled trials of active forms of vitamin D included in the primary analyses and sensitivity analysis.**

| **Source** | **Study Country** | **Treatment** | **Numbe of Participants** | **Age**  **(Mean±SD)** | **Gender (M/F)** | **Dwelling** | **Study**  **Length** | **Change in 25-Hydroxyvitamin D Level in Intervention Group, Mean (SD), nmol/L** |
| --- | --- | --- | --- | --- | --- | --- | --- | --- |
| **Gallagher, 2001** | USA | 0.5 µg 1,25-dihydroxyvitamin D3 | 246 | 72 (3) | 0 /123 | Ambulatory | 3 years | 74.8 (29.0) to 55.5 (20.5) |
|  |  | Placebo |  | 71 (4) | 0 /123 |  |  | 80.5 (27.4) to 63.2 (19.7) |
| **Dukas, 2004** | Switzerland | 1 µg 1α-hydroxyvitamin D3 | 378 | 75.0 ±4.4 | 93/99 | Community dwelling | 36 weeks | 78.0 (21.6) to 60.7 (19.7) |
|  |  | Placebo |  | 75.0 ±4.1 | 90/96 |  |  | 70.8 (26.8) at baseline |
| **Gallagher, 2007** | USA | Calcitriol 0.25 μg twice daily | 213 | 65–77 | 0/101 | Ambulatory | 3 years | Not stated |
|  |  | Placebo |  |  | 0/101 |  |  |  |
